# Emerging Links between Cerebral Blood Flow Regulation and Cognitive Decline: A Role for Brain Microvascular Pericytes

**DOI:** 10.14336/AD.2022.1204

**Published:** 2023-08-01

**Authors:** Tong-Yao You, Qiang Dong, Mei Cui

**Affiliations:** ^1^Department of Neurology, Huashan Hospital, Fudan University, Shanghai, China.; ^2^Department of Neurology, Huashan Hospital, State Key Laboratory of Medical Neurobiology and MOE Frontiers Center for Brain Science, Fudan University, Shanghai, China.; ^3^National Center for Neurological Disorders, Huashan Hospital, Fudan University, Shanghai, China.

**Keywords:** basal tone, blood flow, cognitive impairment, microvascular pericytes, neurovascular coupling

## Abstract

Cognitive impairment associated with vascular etiology has been of considerable interest in the development of dementia. ﻿Recent studies have started to uncover cerebral blood flow deficits in initiating cognitive deterioration. Brain microvascular pericytes, the only type of contractile cells in capillaries, are involved in the precise modulation of vascular hemodynamics due to their ability to regulate resistance in the capillaries. They exhibit potential in maintaining the capillary network geometry and basal vascular tone. In addition, pericytes can facilitate better blood flow supply in response to neurovascular coupling. Their dysfunction is thought to disturb cerebral blood flow causing metabolic imbalances or structural injuries, leading to consequent cognitive decline. In this review, we summarize the characteristics of microvascular pericytes in brain blood flow regulation and outline the framework of a two-hit hypothesis in cognitive decline, where we emphasize how pericytes serve as targets of cerebral blood flow dysregulation that occurs with neurological challenges, ranging from genetic factors, aging, and pathological proteins to ischemic stress.

## Introduction

Continuous cerebral blood flow (CBF) is necessary to meet the metabolic demands of neural function. Accordingly, the parameters of vessels, such as lumen diameters, blood viscosity, microturbulent flow, hemodynamic resistance and shear stress, within the brain are all carefully tuned to match the proper extraction of blood oxygen via a subtle blood flow rate [1]. The CBF rate is maintained at approximately 50 ml/100 g·min during the resting state. The brain extracts 40% oxygen and 10% glucose from arterial blood due to its high energy consumption (20% of the total body oxygen and 25% of the total blood glucose) [2]. This fluctuating matching can be further categorized into the following integrated regulations: perfusion-dependent autoregulation, vascular reactivity to vasoactive stimuli, neurovascular coupling and endothelium-related responses [3].

To date, mounting evidence has identified a temporal-spatial pattern of decreased cerebral blood flow in cognitive impairment and dementia progression [4], indicating that blood flow dysfunction correlates with cognitive injuries in degenerative disorders [5, 6]. Prior cross-sectional studies found a synergistic signature between cerebral blood flow decline and cognitive deterioration after the diagnosis of dementia or related diseases, suggesting the possibility that vascular plasticity may have been involved in ensuring optimized oxygen extraction via adapted counterintuitive suppression of blood flow [7, 8]. However, large population-based cohort studies have contradicted this concept and delineated the sensitive value of cerebral blood flow change in revealing the early potential risk of developing dementia[9], making decreased cerebral blood flow one of the possible determinants of cognitive impairment. Notably, CBF decline originates in the scattered small parts of the posterior cingulate gyrus, precuneus and thalamus before worsening to more extensive areas, including the temporal-parietal lobe [10]. These identified continuums also allow one to speculate whether decreased CBF in a particular region has a localized effect on a cognitive domain.

Considering the tight link between the CBF reserve and intercellular activities of the neurovascular unit (NVU) [11], the role of brain microvascular pericytes has been accentuated in the decline of CBF, potentiating neurodegeneration. Pericytes were originally observed by a French physiologist, Charles Rouget, as a group of cells lying proximate to capillaries with the highest density in the brain [12]. Subsequently, pericytes have been more specifically characterized as having a “bump on a log” appearance [13] with protruding ovoid bodies and long thin processes that reside partially along the microvascular surface, expressing several specific markers, including platelet-derived growth factor receptor β (PDGFRβ) and the proteoglycan NG2 [14, 15]. As a specialized form of mural cells [16] within capillary vessels, pericytes are the only cells with contractile properties that engage in the control of vessel diameters and CBF modulation [17]. Consistent with their structural plasticity, the influence of pericytes on capillary diameters and the quality of flux have been estimated to ensure 84% state-dependent change in CBF [18]. Therefore, dysfunction of pericytes may accelerate cognitive decline by a secondary hit of energy metabolism in the face of the flow decrease. In this review, we discuss the features of pericytes in maintaining an optimized flow pattern and so as to meet metabolic demands. Since the contraction of pericytes could be further affected by coexisting amyloid-β exposure [19] or ischemic attack [20], which would exacerbate the vulnerability of the cognitive status, future research should decipher the complicated interplay among pericytes, the understanding of cognition and the rheological aspects of cerebral circulation.

## Initial blood flow insufficiency promotes cognitive impairment

An adequate blood supply is regarded as a decisive component for brain function maintenance. Anatomically, penetrating arterioles carry blood flow perpendicularly from the cortical surface pia down to the six cortical layers of gray matter, where capillaries branch out to an interconnecting web containing both diverging and converging junctions, discharging blood into the parenchyma, and keeping all red blood cells percolating single file through transit. Capillary perfusions are regulated by switching “ON” and turning “OFF” into different segments. Hematocrit variability in the capillaries determines the fluctuations in the RBC velocity [21], thus redistributing blood flow and RBCs to channel sufficient energy to where they are needed within the network [22]. Then, venule circulation ultimately reunites and drains the capillary blood back [23].

While CBF decreases are related to cognitive decline in normal aging as an independent factor [24], disrupted functional blood supply also provides insight into how dementia develops in several neurologic conditions, such as Alzheimer’s disease (AD) and vascular cognitive impairment (VCI) [25]. Biomarker-related studies suggested that vascular factors, including blood flow reduction, induced the earliest pathological alterations in the 30-year period tracking all possible late-onset Alzheimer’s disease (LOAD) clinical transitions and processes from records of the Alzheimer’s Disease Neuroimaging Initiative (ADNI) database, while Aβ deposition, metabolic impairment, functional changes and structural atrophy were featured in succession [26]. This work redefined the classical temporal evolution of the pathological cascade in Alzheimer’s disease in which CSF Aβ_42_ was dynamically the earliest [27]. Evidence concerning the chronological time frame of genetic frontotemporal dementia also implied a more extensive change in perfusion than in structural modifications during the presymptomatic stages. Longitudinal monitoring revealed an inverse correlation between CBF and the age of expected symptom onset in carriers in this case [28]. In addition, poor cognitive performance was particularly related to a reduced regional blood volume in new cases of Alzheimer’s disease; among these, the precuneus was thought to be the first to show hypoperfusion 10 years before symptoms developed [6].

Following the line of reasoning for VCI, the key etiological processes primarily occur at the level of the blood vessels. Ischemic damage featured an early disruption to the brain’s vascular supply. In particular, hemodynamic lag in stroke patients correlated with neural function and clinical deficits [29]. Microinfarcts have been thought to result from the loss of small vessel flow; in turn, they cause persistent attenuation of evoked hemodynamics and neuronal activity over areas larger than the lesion core, likely lowering the threshold for cognitive impairment [30]. Substantial evidence links impaired CBF to an increased risk of cognitive dysfunction in individuals with vascular risk factors (VRFs). Considered as one of the leading causes of age-related cognitive impairment, hypertension is strongly associated with reduced cerebral perfusion [31]. While global CBF was largely preserved in type 2 diabetes mellitus (T2DM) patients relative to controls, regional hypoperfusion mainly occurred in the occipital lobe, temporal lobe, precuneus, ﻿posterior cingulate cortex and cerebellum, which were associated with poorer cognitive performance in corresponding domains [32-34]. Similarly, the degree of aortic stiffness was negatively related to the cerebrovascular reserve [35]. These studies emphasized the concept that a decrease in CBF was more of a primary output of dedicated pathophysiological processes during the cognitive decline than a final consequence of whole-brain dysfunctions [26].

The hippocampus (HC) has a lower capillary density and narrower diameters than the primary visual cortex (area V1), leading to a lower resting blood flow and oxygenation [36]. Given the well-documented significance of the HC in episodic memory, declarative memory, spatial navigation and other cognitive domains [37-40], lower CBF in the hippocampus itself is associated with impaired cognition [21]. The mixed hippocampal blood supply by the anterior choroidal artery (AChA) and the posterior cerebral artery (PCA) in at least one hemisphere establishes a vascular reserve balance for structural integrity, especially being less subject to atrophy compared to the single supply by the PCA [21], as well as other positive effects in the medial temporal lobe (MLT)-related cognitive domain. In other words, a putative decrease in perfusion may result in higher susceptibility towards HC-related cognition, largely consistent with the vulnerability of the hippocampal cells after hypoxic insults. Therefore, dysregulation of cerebral blood supply could largely limit the efficacy of substrate delivery and give rise to subsequent cognitive deterioration, whereas therapeutic strategies targeting CBF preservation may hold promise in preventing cognitive dysfunction.

## Cellular control of cerebral blood flow: potential machinery in pericytes

Blood flow constantly fluctuates across the brain, and an interplay of sensing and signaling across several cell types in the NVU coordinates the tight regulation of CBF. Although CBF is, of course, controlled both by the adjustments of the arteriole and the capillary level, the majority of hydraulic pressure lies in the capillary bed [23]. Brain microvascular pericytes lodge on the abluminal side of endothelial cells and lie inside the astrocytic end feet [22]. They coordinate blood flow appreciably in a given region with neuronal demand through the capillary vasculature. Controversies have arisen regarding the extent of regional CBF modulation due to smooth muscle cells or pericytes in physiological and pathological vasomotion [41]. Pericytes appear as spatially isolated cells on the capillary [14] compared to the continuous wrapping of contractile smooth muscle cells in an arteriole. Nonetheless, the pericytes’ function is presumably centered on modulating vascular blood flow [42] rather than reflecting a merely passive response to an increasing pressure from dilating arterioles[18], as evidenced by the expression of contractile molecules, such as myosin heavy chains 9/10/11, myosin light chains 6/9/12a/12b [43] and regulators of myosin phosphorylation state [44] by single-cell RNA-seq and other transcriptomic analyses. Studies investigating rodent brain slices first demonstrated that pericytes could alter capillary diameters in response to neurotransmitters, electrical stimulation and ischemia ex vivo [17]. Neighboring pericytes also negotiate their territories via continuous activity of terminal processes, forming a complementary, nonoverlapping chain-like regulatory network along the entire capillary bed [45].

The integration of pericyte/endothelial communication instantiates the converging points of diverse functional responses in pericytes, including the regulation of capillary hemodynamic responses, angiogenesis, neuroinflammation, clearance of toxic metabolites, stem cell activity and BBB permeability [16], which contribute to maintaining CBF for microvascular stabilization. Pericytes share the common basement membrane with capillary endothelial cells, allowing for elaborate cell-to-cell signaling. The microcirculatory vasodilating effects of endothelial C-type natriuretic peptide (CNP) are mediated by guanylyl cyclase-B (GC-B)/cGMP signaling in pericytes, moderating calcium responses to endothelin and regulating the peripheral vascular tone [46]. Intriguingly, pericytes stabilize vessels as their contractility regulates endothelial angiogenic behaviors [47]. The expression of vascular endothelial growth factor receptor 1 (VEGFR1) in pericytes spatially restricts vascular endothelial growth factor (VEGF) signaling [48]. Subsequent to the detachment of pericytes, specialized tip cells in the endothelium initiate the migration and outgrowth of blood vessels towards gradients of vascular endothelial growth factor A (VEGF-A) in the early stages of sprouting [48]. Nascent vessels, in turn, recruit pericytes to the vessel walls through ﻿platelet-derived growth factor B (PDGF-B)/PDGFRβ signaling, during which attached pericytes stabilize tight junctions, decrease transcytosis via vitronectin-integrin interactions [49], and suppress the expression of leukocyte adhesion molecules in the endothelium to reduce the invasion of peripheral immune cells, further promoting BBB maturation and CNS immune surveillance [50, 51]. Moreover, pericytes facilitate the quiescence of the microvascular endothelium within angiopoietin (﻿Ang)/Tie crosstalk [52]. Consequently, microvascular integrity is maintained by proper interactions between endothelial cells and pericytes, establishing the emerging role of pericytes in CBF regulation.

Due to the contractile nature of brain microvascular pericytes, blood flow through capillaries can be controlled to ensure the energy supply through precise temporal and spatial modulation of regional CBF. First, the morphology of pericytes and their suitable location in different segments provide partial clues regarding their control over vessel diameters, vascular resistance, and blood flow. Pericytes also present a baseline contractile tone directly within the microvessels, exerting broad forces capable of executing vessel diameter changes on both their bodies and processes. Similarly, the role of pericytes in neurovascular coupling (NVC) emphasizes the potential for how upstream changes in neuronal activities could alter the blood flow balance in the entire brain. Here, we elaborate upon three mechanisms to discuss the role of pericytes in normal CBF regulation.

### High regional heterogeneity of pericytes towards structural network remodeling

The soma of pericytes is preferentially stable in the location of capillary junctions, while its elongated processes are more active, with variable extensions and retractions occurring within days to weeks [45]. In normal adult mouse brains, blood flow and the red blood cell velocity decrease nonlinearly throughout the capillary network, as the first few successive junctions adjacent to feeding arterioles constitute the primary sites of flow resistance; thus, pericytes situated in these transitional regions might display an outsized influence on this distribution heterogeneity [1, 53, 54]. Both ex vivo and in vivo evidence implies that junctional pericytes are potentially positioned to influence the capillary diameters and to compensate for the additional resistance imposed by the original symmetry of bifurcations [13]. Accordingly, there is a stepwise evolution of brain microvascular pericytes in terms of morphology, protein expression and contractility as the branch stage increases along the capillary bed [55]. The first branch from the main penetrating arteriole trunk lacking an internal elastic lamina is considered as the 1^st^ order offshoot of capillary segments, and then, the order increases with each subsequent bifurcation regardless of the size [13]. The ovoid cell bodies of pericytes can be observed at the 1^st^ order, and α-SMA expression terminates at the 3^rd^ to 4^th^ order. The vessel coverage shifts at the 5^th^ to 6^th^ order, followed by an expanded inter-soma distance as vessel diameters decrease. These transitions occur more obviously on smaller penetrating arteriole branches. Only pericytes limited to the 1^st^ to 3^rd^ junctions within the capillary network show positivity for the contractile isoform of actin—α-actin (Acta2) [13], which could impact on the gate of blood flow. A higher expression of α-SMA in pericytes on first-order capillaries also contributes to an increased contractile capacity, bearing more pressure than above and below [11].

In short, microvascular pericytes are generally subcategorized into the following three subgroups [55]: 1) ensheathing pericytes, which are upstream of the α-SMA terminus (i.e., 1^st^ to 4^th^ -order branches) with dense wraps around the capillary segments and a more circumferential morphology; 2) mesh pericytes, which have a more longitudinal process in the middle of the capillary bed and an intermediate coverage of the vessel, but they lack α-SMA expression and a prominent banding pattern; and 3) thin-strand pericytes, which can be referred to as stellate-shaped cells with thinner processes and fewer projections along the capillary for longer distances [56, 57]. The latter two subclasses are also defined as capillary pericytes together due to α-SMA absence [15]. In the HC there are no morphological differences between ensheathing pericytes and their covering vessels with the cortex, but they show smaller and less frequent dilation when they respond. Mesh and thin-strand pericytes have longer processes in the smaller vessels they are tantamount to follow [36], suggesting lower contractility since vasomotion near the soma is much stronger [58].

There are emerging aggregation sites of vascular conductivity to promote vasomotion signals in the brain. Alarcon et al. recently identified interpericyte tunneling nanotubes (IP-TNTs) that had a soma-to-process connection via direct membrane-to-membrane contacts to separate capillary pericytes in the mouse retina [59]. Each IP-TNT was a single process containing organelles, including mitochondria and the endoplasmic reticulum. IP-TNTs mediate bidirectional intercellular Ca^2+^ waves, local ATP production and vesicle trafficking via connexin 43-based gap junctions. Their connected capillary pairs always underwent coordinated opposite responses after light stimulation. One dilated to increase perfusion, while the other constricted to reduce blood flow. These activities were highly correlated with contrary and synchronized Ca^2+^ transients in corresponding pericytes. IP-TNT ablation simultaneously reduced the total Ca^2+^ transient frequency in coupled pericytes, and linked capillaries exhibited severely compromised harmonized dilation and constriction elicited by light and accommodated blood flow later [59]. Similarly, the enrichment of “peg-and-socket” interactions between pericytes and endothelial cells also played a role. The presence of claw-like pegs/columnar protuberances anchored to the endothelium along with a multitude of adhesive junctions allowed pericytes to exhibit and project translating force securely into the underlying endothelium during contraction or relaxation [60].

### Pericytes regulate the basal vascular tone in homeostasis

Nearly one-third of capillaries showed an increase in flux, one-third underwent a decrease and one-third remained unchanged under basal conditions [22], where neuromodulators, such as serotonin (5-HT), noradrenaline (NA) and myogenic tone promote widespread vasoconstriction together. Pericyte-mediated capillary tone could already be established during the angiogenic process. In mouse neonates, sprouts had a tendency to emerge from ascending venule bifurcations where the pericyte soma was located, and then, they diverged to their targets in penetrating arterioles or immediate offshoots, which is consistent with the fact that pericyte somas stayed more prevalently at capillary branches in the adult brain [61]. These new sprouting capillary diameters were pressurized during blood cell perfusion and stabilized with myogenic tone through consistently reduced diameters [62].

One of the most prominent effects of pericytes have on the development of the basal capillary tone is related to their structural plasticity. Microvascular pericytes do have spontaneous microdomain Ca^2+^ transients within their processes. These dynamic microdomain Ca^2+^ elevations partially sustain the resting Ca^2+^ level in pericytes and the basal tone of microvessels [63]. Ca^2+^ removal and glutamate application dilated cerebellar capillaries by decreasing tone near pericytes[64]. Each pericyte constituted a restricted Ca^2+^-signaling domain and controlled the diameters of separate functional branches independently [13], while stimulated Ca^2+^ events in pericytes in the proximal transitional region could conduct a Ca^2+^-dependent contraction in the projections of distal pericytes, increasing their average contraction frequency [13]. Therefore, pericytes structurally modulate the resting geometry of capillary branches. Uncovered capillary segments in the brains of mice were more dilated due to a reduced constriction ability after single pericyte ablation by two-photon irradiation, suggesting the immediate loss of tone as opposed to long-term remodeling by the endothelium. However, neighboring pericytes could actively adapt to extend their processes and recover the exposed endothelium for entire contact during the next few weeks. Thus, the capillary lumen diameters regained the former state of vasoconstriction [45].

Interestingly, smaller-diameter capillaries responded with greater dilation after pericyte ablation than larger capillaries, raising the possibility that variable levels of tone are controlled by pericytes. Pericytes tended to exhibit a higher frequency of Ca^2+^ events when encircling the smaller-diameter corresponding segments [13]. Both optogenetic ChR2-YFP stimulation and transient hypercapnic challenge of brain capillary pericytes caused a gradual decrease in the lumen diameter with blood flow on a slower timescale than upstream ensheathing pericytes in vivo, and preceding diameter changes than RBC velocity. They were also slower to dilate back following vasoconstriction [15]. These slow kinetics further indicate that capillary pericytes maintain flow resistance with heterogeneity at rest, in contrast to the rapid modulation required for vasoactive stimuli by ensheathing pericytes [13, 63, 65, 66]. These heterogenous capillary diameters and flow rates under basal conditions provide a reserve for perfusion changes and enabled better oxygen delivery [67, 68]. More specifically, ROS are generated by mitochondrial calcium uptake due to increased calcium influx[69], leading to Rho-kinase activation in pericytes and underlying vasoconstriction [70] rather than nonspecific passive mechanisms.

### Pericytes promote vasomotor responses during neurovascular coupling

Neurovascular coupling, or functional hyperemia, is a robust process patterned by the synchronous work of cells within the NVU spatially and temporally [58], where active neurons dilate local blood vessels, increasing blood flow and oxygen/glucose supply to those vibrant regions through glutamate and nitro oxide (NO) signaling [36, 71]. The activity of microvascular pericytes has been regarded as an accurate temporal marker of synaptic activation during neurovascular coupling [63]. Pericytes play an active role in capillary relaxation and blood flow heterogeneity modulation, reflecting a parallel increase in capillary structural elasticity and coordinated energy supply [72]. To study NVC in vivo, sensory stimulation can be processed into the barrel cortex in awake mice upon whisker brushing, evoking well-characterized neural activities in the mouse somatosensory cortex with intracellular calcium level changes [73].

Capillaries detect and dilate earlier than arterioles during neurovascular coupling. They dilated approximately 1 s before arterioles in mice after electrical stimulation on the whisker pad, reaching a larger magnitude and higher frequency in areas covered with pericytes [18, 74]. Neuronal activities evoked an outward membrane current in pericytes by local neurotransmitter release. In response, these associated pericytes then sensed and changed the resting tone they set to be faster than smooth muscle cells. Therefore, capillaries were able to send back vasodilatory hyperpolarizing signals to arterioles [17]. The direct administration of ATP into the mouse somatosensory cortex also led to synaptic activation with the earliest and strongest functional dilation in the 1^st^ and 2^nd^ capillary branch order near pericytes, followed by upstream and downstream propagation linearly at 5-20 µm/s.

The entire capillary bed was able to redistribute the blood flow and channel red blood cells within pericyte-mediated dilation after the onset of functional hyperemia in mice [22]. Normally the largest proportional velocity increase occurs in the juxta-synaptic capillaries, and pericyte-mediated dilation could occur throughout at least the fourth branch order and propagate between adjacent pericytes [17]. However, pericytes in discrete regions lead to different capillary flow rates, improving the coordination and cooperation between successive processes. For example, pericytes in the 1^st^ -order branch drive a robust dilation but a lagging increase in the RBC velocity, contrasting those in the 2^nd^ to 6^th^-order branches, where a slower dilation and rapid velocity change occur [63]. Ca^2+^ transients in thin strand pericytes could not evoke the onset of increasing RBC velocity. A wider range of capillaries display a rise in flux at the expense of some exhibiting a decrease; however, both underwent an equal 10% diameter change, and this alternation was sufficient to govern RBC passage during dilation and constriction [22].

Several pathways are involved in pericytes during NVC; among these, the K_ATP_ channel serves as a key contributor. Due to the prevalence of K_ATP_ channels in brain pericytes, branch-specific dilations within individual enwrapping projections occur after receiving K^+^-dependent propagating hyperpolarizing signals at the site of neuronal activities [13]. Following glutamate release, prostaglandin E_2_(or a related species activated by EP_4_ receptors) is generated to mediate an outward K^+^ current in pericytes. Although noradrenaline generated 20-HETE to constrict pericytes, the release of nitric oxide (NO) can suppress 20-HETE formation [18]. Potassium outflow and membrane hyperpolarization were associated with the closing of L-type Ca^2+^ channels to reduce intracellular Ca^2+^ [75]. Thus, K^+^-induced vasodilation enhanced the NVC response, directed blood flow and asymmetric structure along the daughter branches, paving the way for the dynamic guidance of junctional blood flow to favor the flux of RBCs towards the stimulated region [15].

Purinergic signaling cascades also play a role. ATP released by postsynaptic neurons can act on astrocyte ATP-gated channels (including P_2_X_1_ subunits) and conduct a [Ca^2+^]i influx signaling after upstream neuronal activity. Therefore, astrocytes generate arachidonic acid via the phospholipase D_2_-diacylglycerol lipase (PLD_2_-DAGL) pathway, followed by COX1 metabolism to produce PGE_2_ and dilated capillaries through the EP_4_ receptor in pericytes[76]. In contrast, glutamate-evoked NO release from interneurons dilated arterioles in an NMDA receptor-dependent manner, revealing that astrocytic Ca^2+^ signaling was sensed by capillary pericytes rather than arterioles. Furthermore, endothelial-derived epoxyeicosatrienoates (EETs) facilitated the relaxation of pericytes during NVC, while the overexpression of soluble epoxide hydrolase (sEH, EETs metabolizing enzyme) in endothelial cells constricted pericytes and terminated the dilation [22].

## Pericyte dysfunction accelerates cognitive impairment *via* decreased CBF

Cognitive injury secondary to cerebral hypoperfusion within capillaries seems persistent but largely underrecognized. For instance, the HC sustains neuronal function through increasing oxygen extraction from slower-moving RBCs, despite the lower vascular density and weak contraction abilities in pericytes. During NVC, neuronal activity is generally restricted by mismatched oxygenation supply due to the limited ATP synthesis caused by a combination of reduced blood oxygen saturation and increased capillary spacing in the HC. There is a larger volume of vulnerable tissue under hypoxic conditions in the HC than in the neocortex when CBF decreases [15], resulting in a higher risk of focal cognitive impairment.

**Figure 1.**
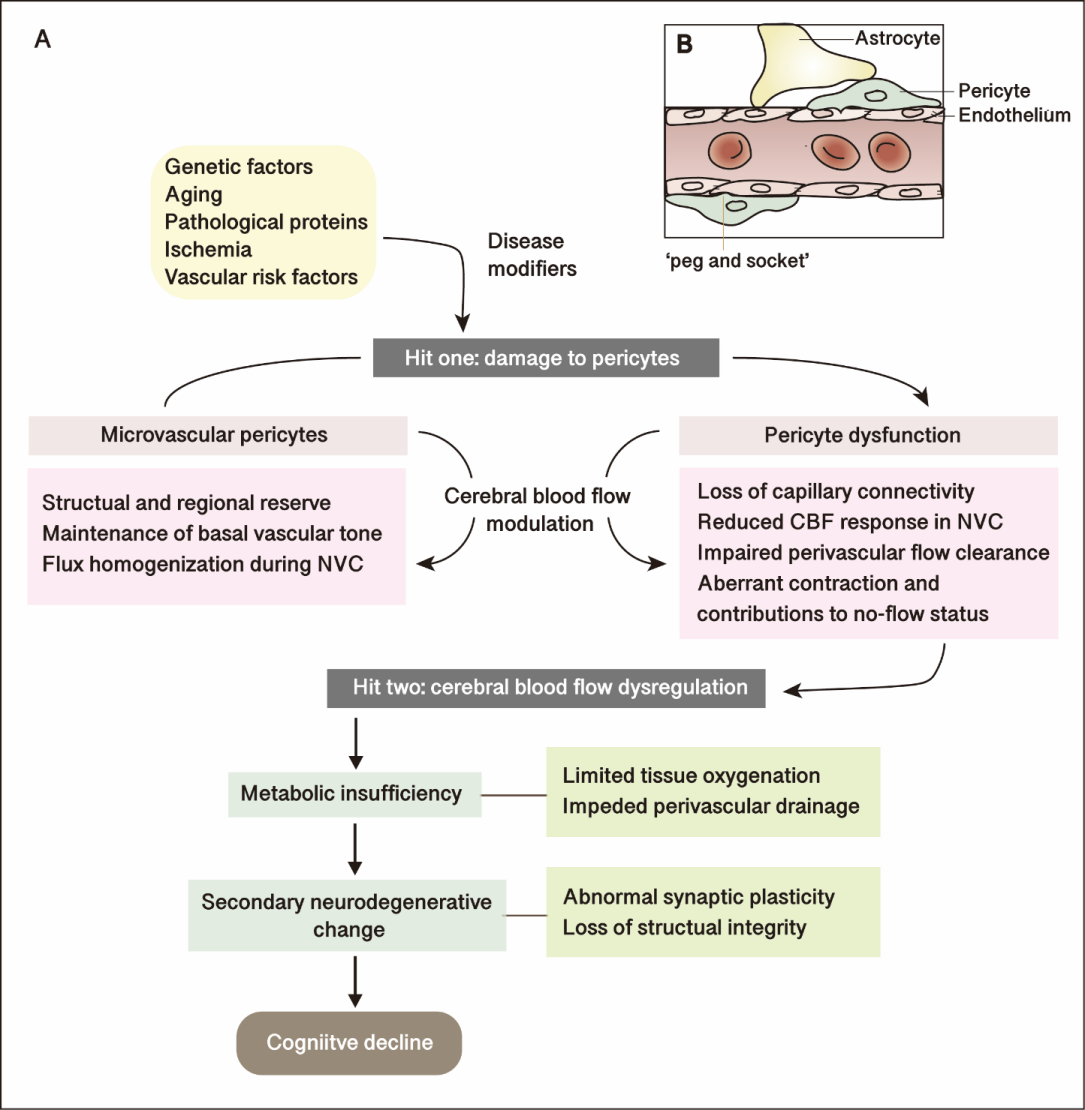
**Pericyte dysfunction in cognitive decline: two-hit hypothesis through cerebral blood flow modulation**. (**A**) Schematic summarizing effects of risk factors along with disease modifiers lead damage to microvascular pericytes (hit one), which disrupt the natural modulation of cerebral blood flow by pericytes in relation to structural integrity, vascular tone and flux homogenization. Functional blood flow supply in response to pericyte dysfunction deteriorates (hit two), followed by consequences of metabolic changing and structural injuries to the potentials of cognitive decline. (**B**) Schematic representation of a longitudinal section through the capillary. Endothelial cells tightly form the vessel walls. Pericytes are mural contractile cells located in microvascular vessels, with ovoid-like bodies and stretching processes. Pericytes contact with endothelial cells along the shared basement membrane, underscored by the ‘peg-socket’ interdigitations. Both pericytes and endothelial cells are covered by astrocytic endfeets. Abbreviations: NVC, neurovascular coupling; CBF, cerebral blood flow.

Broadly, the mechanisms underlying the CBF deficits in the cognitive deterioration remain poorly understood since the origin is likely multifactorial, and the role of pericytes in cognitive decline has not been thoroughly illustrated. Recently, the ratio of myelin-associated glycoprotein to proteolipid protein-1 (MAG:PLP1, which represents chronic hypoperfusion) and the PDGFRB levels were shown to decline early with a positive correlation in the precuneus from post-mortem tissue of Alzheimer's disease and age-matched control brains[77]. Accelerated cognitive and pathological deteriorations were also noted in Pdgfrβ^+/-^ mice crossed with AD-related transgenic mice [78]. Indeed, all the impaired biological features of pericytes are thought to be involved in the evolution of cognitive impairment, such as BBB maintenance, angiogenesis, and CBF regulation. Delayed capillary dilation and decreased RBC velocity in response to neuronal stimulus strongly suggest that pericyte dysfunction serves as an ongoing factor in neurovascular uncoupling in 1-to 2-month-old pericyte-deficient mice (PDGFRβ^+/-^ mutants) [58]. Considering that all CNS cells need a matched CBF supply, we propose a two-hit hypothesis focusing on pericyte dysfunction in cognitive decline through cerebral blood flow modulation. Classic pathophysiological contributors could serve as a key point in initiating pericyte deterioration (hit one) and, thus, driving CBF stagnation (hit two), which may subsequently be exacerbated by the dysfunction of nearby associated cells, aggravating energy deficits and cognitive decline (Fig. 1). Under these conditions, microvascular pericytes would undergo early degeneration and develop an impaired hemodynamic response, while neuronal excitability and other cells in the neurovascular unit remain intact. Consequently, the reduction in the oxygen supply to the brain exists both at rest and after a stimulus, and lactate released from astrocytes becomes a preferred energy source for activated neurons [79].

### Genetic background

Apolipoprotein E (APOE) is expressed in multiple brain cell types and acts in synergy with accumulated vascular defects to affect cognition. There are 3 alleles of mutants of the human apolipoprotein E gene, namely APOE ε2, APOE ε3 and APOE ε4. The APOE ε4 allele remains the strongest genetic susceptibility factor for sporadic AD and other vascular conditions. Interestingly, APOE genotype alters CBF. APOE ε4 carriers show faster decreased CBF after middle age during normal aging[80]. Global Aβ-independent CBF reductions are more widespread in APOE4 carriers, and these changes are not restricted to typical regions showing early hypometabolism in AD [81]. In APOE4-targeted replacement mice, there is a reduction in cortical CBF, followed by greater perfusion deficits and reduced post-synaptic density in longitudinal analyses [82]. Single-cell RNA sequencing revealed that the conditional expression of ApoE4 in vascular mural cells (VMCs) led to reduced CBF, compromised synaptic plasticity and spatial learning in mice, accompanied by astrocyte activation [83]. Given that elevated baseline CSF levels of soluble PDGFRβ (sPDGFRβ, a marker of injured pericytes) have been postulated to predict future cognitive decline in APOE ε4 carriers [80], we suppose that APOE could differentially modulate CBF and cognitive potentials in a pericyte-dependent manner.

Prior studies have considered pericytes a key modulator of the proinflammatory CypA-nuclear factor-kB-matrix metalloproteinase-9 pathway by different APOE isotypes. Astrocyte-secreted APOE3 and murine APOE had a high affinity for pericytes via the LPR1 receptor, leading to suppressed CypA synthesis, whereas cerebral pericytes in Apoe-deficient and APOE4 mice exhibited the opposite proinflammatory response and correlated well with reduced regional CBF and BBB breakdown [84]. A greater pericytes loss was observed in APOE4 lines than in APOE3 lines independent of Aβ pathology [85], which deprived neurons of pericyte-derived neurotrophic support in the presence of diminished CBF, such as pleiotrophin (PTN) [74]. PTN loss further renders neurons vulnerable to circulatory stress, like BBB disruption, vasogenic edema and blood flow reduction, accelerating neuron loss and behavioral deficits. The greater BBB breakdown in AD APOE4 carriers has been partly related to accelerated pericyte degeneration compared with APOE3 carriers since chronic BBB disruption has been linked to small vessel diseases and AD [86]. Of note, Casey et al. demonstrated that APOE4 in pericytes led to an enhanced migratory phenotype in a RhoA-mediated manner [87]. APOE4-specific pericytes were also less efficient in supporting basement membrane formation and endothelial barrier integrity [88]. It is possible that APOE4-regulated pericyte migration contributes to the imbalance of angiogenesis and disturbs normal BBB function in the pathogenesis of cognitive impairment.

In the human brain of APOE4 carriers and the induced pluripotent stem cell-based three-dimensional APOE4/4 BBB model, calcineurin-nuclear factor of activated T cells (NFAT) signaling was selectively dysregulated in pericytes, interacting with the APOE promoter and upregulating the APOE protein levels to amyloid accumulation, illustrating the pathogenic effect of APOE4 in cerebral amyloid angiopathy (CAA) [89]. These amyloid deposits impaired the vasomotion of blood vessels and disrupted perivascular flow clearance, leading to increased diffusion of perivascular Aβ and comprising the vulnerable CA1 region of the hippocampus with allocortical microinfarcts, which is known to cause cognitive defects [90].

### Aging

The prevalence of dementia significantly increases with age[91]. Aging is deemed to yield pervasive effects on neurovascular units including pericytes, as evidenced by the significant loss of pericyte numbers(more than 20%) and coverage(almost 50%) of capillaries, along with dramatically downregulated genes, such as Pdgfrβ, Act2, and Cav2, in aged mice [92]. Throughout the temporal sequence of events, neuronal injury and neurodegenerative changes could come down to the degeneration of vascular pericytes, which begins with CBF insufficiency, followed by chronic hypoxic stress.

There was a modest 20% decrease in pericyte coverage in young Pdgfrβ^+/-^ mice (1 month old) as vascular injuries, like diminished perfused capillary length and CBF response, were well established [93]. This primary vascular damage preceded profound neuronal injuries. Studies have shed light on the remodeling capacity of pericytes to fill incomplete gaps in lower branch capillaries by extending their processes when there was a 25-30% decrease in number[94], which may account for the substantial microvascular flow rates at this time point in the absence of neuronal changes. However, pericyte-specific insults resulted in apparent hemodynamic disturbances in the neuronal structure and cognitive function in Pdgfrβ^+/-^ mice at 6-8 months [93].

Pericyte remodeling was less efficient in the aged brain, causing regional capillary dilation especially in sparser capillary networks such as hippocampus and white matter[36, 95]. However, uneven increased blood flow was generally observed at the epicenter of dilated capillaries, leaving the affected capillaries in the surrounding area decreasing in flow regardless of the extent of dilation. This abnormal partitioning of blood cell flux at bifurcations promoted flow stalling, where blood steal by an adjacent dilated capillary, resulting in an enduring loss of capillary connectivity [96]. The broadening of capillary flux heterogeneity by age-related pericyte degeneration created a larger barrier to flux homogenization, limiting tissue oxygenation at rest and during functional hyperemia. Furthermore, the process of pericyte remodeling contributes to endothelial coverage and vascular tone, Eventually, the brain’s ability to properly allocate energy for cognitive learning was disrupted, followed by string capillaries, and overall reduced capillary density in aging and dementia. Taken together, Pdgfrβ^+/-^ mice exhibited a quantitative relationship between age-dependent advanced pericyte loss and reductions in the brain capillary density, resting CBF, and CBF responses to neuronal activation. Moreover, the cascade of vascular, neuronal and behavioral patterns was more pronounced in F7/F7 mice carrying seven point mutations in PDGFRβ with greater pericyte loss [97].

The question that remains is whether perfusion stress alone is responsible for age-dependent microvascular degeneration. While BBB integrity remained within a physiological range in VMC-specific APOE4 mice, chronic hypoperfusion could contribute to cognitive decline. In contrast to pericyte-deficient mice, Meox2^+/-^ mice (with a single deletion of the mesenchyme homeobox gene-2 allele) at a comparable age exhibited a significant perfusion deficit with 45%-50% reductions in the capillary density and resting CBF but normal pericyte coverage of microvessels [98]. Notably, Meox2^+/-^ mice maintained unaltered hemodynamic responses to stimulation, while Pdgfrβ^+/-^ mice were much more marked in impaired CBF response and neuronal injury, confirming that ﻿perfusion stress and BBB compromise of pericyte dysfunctions are associated with ﻿impairments in memory and learning mediated by neurovascular insults [93]. Diminished brain microcirculation led to chronic perfusion stress and hypoxia, along with accumulated neurotoxins secondary to BBB breakdown to induce greater neurodegeneration, while intact neuronal circuitries mediate cognitive behavioral responses. Moreover, in aging, brain endothelial PDGF-B expression was impaired [99], and without PDGF-B recruitment of pericytes, BBB leakage could not be easily rescued [100].

### Pathological proteins

Over the past years, extracellular amyloid plaque accumulations in the brain parenchyma have been regarded as a classic pathological hallmark of AD [97] and occur long before disease onset [101]. However, several lines of evidence indicate that the levels of amyloid-β peptides (Aβ) do not affect the severity of cognitive dysfunction [102, 103]. Aβ has been proven to impair vascular activity in the development of both chronic vasoconstriction and CAA as indicated by a reduction in cerebral circulation, which ultimately mediates the onset of white matter damage and impairs cognition [26]. Damaged pericyte processes were found to be an early event in Tg-SwDI mice in the CAA model, along with a waned CBF response to whisker stimulation [104]. It is also known that perfusion insufficiency can promote Aβ accumulation [105], as regional perturbation of CBF by transverse aortic constriction (TAC) surgery initiates a global increase of amyloid angiopathy and plaque load [106]. Thus, pericyte dysfunction in blood flow regulation may provide a standpoint for the transition from Aβ deposition to deteriorating cognitive performance.

Pericytes are highly susceptible to Aβ, and CAA can induce the intracellular aggregation of Aβ in microvascular pericytes, triggering cellular stress and death [16, 107]. Post-mortem brain tissue studies verified that the capillary diameters were fairly narrowed near the pericyte soma with Aβ deposition, exhibiting a strong positive correlation between Aβ-dependent pericyte degeneration and the diameters of perfused microvessels. Much of the insight above emphasizes the idea that Aβ probably leads to capillary constriction specifically at pericytes, thereby contributing to subsequent hypoperfusion and impaired vascular clearance of Aβ prior to cognitive defects [19]. In particular, amyloid β oligomers can activate NADPH oxidase and generate reactive oxygen species (ROS) through NOX4 in pericytes [108, 109], which, in turn, evokes the release of endothelin-1. By acting on the ET_A_ receptor in pericytes, the endothelium potentiates the constricting effects of capillaries. In fact, a 30% constriction in pericytes led to a major reduction in CBF, inducing unfavorable feedback towards both basal circulation and a reduction in the flow increased by functional hyperemia. Therefore, energy deficits in the brain persist via the constricting action of Aβ on pericytes. Upon chronic exposure to Aβ, the endothelin-mediated contraction towards pericytes was greatly reduced despite the elevated level of endothelin-1 in the cerebral cortex[110], since amyloid potentially impaired their full effect on pericytes. Consequently, the brain becomes trapped in a vicious cycle of elevated Aβ production through β-amyloid converting enzyme (β-secretase 1, BACE1) activation [111]. Oxidative stress induced by Aβ was thought to be responsible for this moderate injury at an early stage, while functional and structural vasomotor alterations later failed to sustain all vascular responses in aged mice[104].

Evidence suggests factors other than pericyte degeneration could account for the vasoconstriction and neurovascular uncoupling influenced by the Aβ levels. Hypoperfusion of the precuneus in AD was associated with the loss of the pericyte protein PDGFRβ. Conversely, reduced oxygenation of the underlying white matter was correlated with increased fibrinogen in the absence of PDGFRβ change [101]. Aβ_1-40_-induced oxidative-nitrosative stress disturbed vascular autoregulation by activating sustained transient receptor potential melastatin-2 channel (TRPM2) currents in brain endothelial cells, resulting in large increases in intracellular Ca^2+^[112]. Amyloid could also promote arteriolar rigidity and infarction in the territory of their branching vessels through the development of CAA. Alternatively, hypoperfusion along with reduced vascular pulsation further impeded the perivascular drainage of Aβ, forming another cycle of disturbed Aβ metabolism and cortical microinfarcts [113]. Moreover, amyloid accumulation was associated with impaired structural integrity in WMHs, putatively contributing to more pronounced demyelination and axonal damage in the pathological process occurring in AD [114]. In brief, amyloid deposits reduce the dynamic modulation of the NVU, including pericytes, in affected vessel segments and hindered neurovascular functions during dementia progression.

### Ischemic stress

The progression of cerebrovascular pathology in vascular or post-stroke cognitive impairment (PSCI) has greatly advanced our understanding of the vulnerability of brain functions in the setting of acute/chronic hypoperfusion. While endothelium is thought to play a key role in neurovascular injury after ischemia, pericytes are more fragile and sensitive. Pericytes have been shown to be an instant victim after ischemia, developing progressive contraction and swelling [115] with persistent severe constriction of capillaries both in vivo and in brain slices, usually at locations within 1^st^ and 2^nd^-order capillaries [65]. Pericytes died approximately 40 min after their contractile compensation [18], thereby producing a long-lasting resistance in the capillary bed with a prolonged decrease in microvascular blood flow and subsequent no-flow phenomenon.

The aberrant contraction and death of pericytes during ischemia and reperfusion are mainly caused by Ca^2+^ overload through Na^+^/Ca^2+^ changes via a purinergic type 2 receptor-dependent mechanism [65]. There was a robust, sustained Ca^2+^ increase in IP-TNT that markedly impeded the dilation in connected pericytes, which may partly explain the enlargement of hypoperfusion and metabolic disturbances beyond the ischemic zone [59]. The Ca^2+^-gated Cl^-^ channel TMEM16A in pericytes amplified ischemia-evoked-depolarization, and led to a stronger pericyte [Ca^2+^]i rise than store-released Ca^2+^ alone, further evoking profound capillary constriction and favoring neutrophil and platelet stalling [116]. Rho kinase activation also strengthened myosin light chain phosphorylation in pericytes [117] with enhanced contraction. Oxidative-nitrative stress from microvessel walls equally tended to block the restoration of excessive raised [Ca^2+^]i[118]. With a constricted energy supply in the brain, the lack of ATP further hinders myosin and actin from separating, forcing pericytes to arduously cope with ischemic stress.

As a result of postischemic endothelial swelling and pericyte-mediated narrowing of the capillary lumen, leukocytes and platelets stalled in capillaries and contributed to no reflow. In turn, pericyte death induced the expression of leukocyte adhesion molecules and decreased the extent of the cerebral endothelial glycocalyx, which resulted in exacerbated neutrophil plugging of capillary segments with increased leukocyte-endothelial cell interactions [119]. Each occluded capillary reduced CBF in both up- and downstream vessels, resulting in an outsized area of neuronal hypoxia and loss [120]. Collectively, these findings established a link between pericyte damage and the uncoupling of the NVU, wherein capillary stalling is a key facet of the energy deficits. Moreover, no-reflow status hampers tissue recovery even after recanalization, depriving the brain of oxygen necessary for neuronal function, as the loss of hemodynamic rescue should be considered for further synaptic loss and neuron death to memory impairment. Under chronic ischemic insult, the coverage of capillary pericytes in the rodent brain was substantially blunted, followed by white matter degeneration [121]. As a result, mutual effects between perfusion and pericyte function were observed in association with impaired cognition.

### Vascular risk factors

Recent studies have linked pericyte changes over cognitive decline to a few potential mechanisms under VRFs, including hyperglycemia and hypertension. Pericytes are particularly sensitive to these injuries and vascular pathology occurs at early stages. Distinct morphologic changes were observed in pericytes with bridge formation and off-vessel migration in early hyperglycemia, which may explain the increase in pericyte death and CBF compromise [122]. ﻿Glucagon-like peptide-1 (GLP-1) receptor expression and oxidative stress in pericytes were increased in response to chronic hyperglycemia, leaving the pericyte partially off vessel with reduced viability, likely eventually leading to cerebrovascular pathological neovascularization and the loss of vascular protective perfusion [123]. Similarly, hyperglycemia induced the overexpression of angiopoietin 2 (Ang2) in endothelial cells, which aggravated apoptosis in pericytes via the integrin receptor in diabetic retinopathy, whereas Ang2 alone did not induce apoptosis under normal glucose[124].Consistent with compromised interpericyte communication by IP-TNTs, a significant reduction in the capillary diameter and capillary blood flow at pericyte locations was detected early after ocular hypertension (OHT) [125]. Impaired IP-TNTs hindered the rapid relocation of blood supply to those demanding neurons, thus sensitizing neurons to pressure-related stressors. Excessive Ca^2+^ influx to pericytes mediated neurovascular deficits, which, in turn, triggered a decreasing Ca^2+^ peak amplitude response and increased decay time in neurons fed by capillaries with a reduced blood flow. Therefore, VRFs are considered among the leading causes of VCI. They are strongly associated with reduced CBF, and further vascular integrity caused by pericyte dysfunction.

## Possible therapeutic approaches targeting pericytes in the future

Nilvedipine has benefited a subgroup of moderate AD patients by improving hippocampal blood flow [126], suggesting that treatment boosting vascular perfusion and oxygen delivery could be an effective approach if instituted early in cognitive disorders. Pericytes are potential targets to counter CBF deficits. On the one hand, restoring blood flow by preventing aberrant contraction may provide a strategy for cognitive improvements. C-type natriuretic peptide (CNP) supplementation reversed the capillary diameter under Aβ insults, largely due to the activation of myosin light chain phosphatase and the blocking of Ca^2+^ release from internal stores in pericytes [19]. The administration of blockers against the ROS generator NOX_4_ and the constricting ET_A_ receptor on pericytes could prevent further Aβ-evoked capillary constriction in brain slices. After SCI, using antagonists of monoamine receptors or the inhibition of AADC enzyme function could provide substantial relief from pericyte-mediated vessel constriction, ultimately improving blood flow and behavior functions [71]. The pericyte-specific deletion of the alpha 1C subunit in L-type voltage-gated Ca^2+^ channels (Cav1.2) protected IP-TNTs and rescued capillary dynamics during ocular hypertensive stress, highlighting the therapeutic potential of the recovery of Ca^2+^ homeostasis in pericytes restoring neurovascular coupling and promoting neuronal survival [125]. Moreover, the contractile machinery of pericytes can be adjusted by a Rho-kinase inhibitor, which disrupts actomyosin cross-bridge cycling [15]. On the other hand, intra-pericyte antioxidation might be considered a pharmacological microvascular intervention. CypA inhibitors reduced perfusion deficits by suppressing the CypA pathway in pericytes, slowing cognitive deterioration in APOE4 carriers [80]. Treatment with GLP-1RA exendin-4 improved pericyte function by reducing diabetes-mediated oxidative and nitrative stress, likely contributing to the maintenance of cognitive function via glucose-dependent pathways [123].

## Conclusions

Generally, a body of evidence suggests that CBF dysregulation is the early functional event preceding cognitive decline in the pathophysiological cascade of neurodegenerative disorders, such as AD and VCI. Our review identifies the role of brain microvascular pericytes in CBF regulation and capillary dynamics modulation during energy-intensive brain activity. Although pericyte dysfunction is not the sole driver of CBF deficits, the aberrant contraction of capillaries produced by pericyte degeneration along with the decrease in CBF under risks leads to neurovascular uncoupling and cognitive deterioration. However, several studies offer new sights for therapeutic interventions targeting pericytes since the reversal of disrupted CBF supply could restore cognitive function if the damage to neuronal synapses and circuits has not significantly advanced. More research could determine the cellular mechanisms of pericyte degeneration that translate to CBF changes in the future, and how several dysfunctions of pericytes could interact with other cell types and contribute to cognitive impairment remains unclear.
